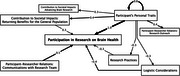# Gender‐Related Facilitators and Barriers to Participation in Observational Neurocognitive Aging Research in Older Adults: A Fuzzy Cognitive Mapping Approach

**DOI:** 10.1002/alz70857_096634

**Published:** 2025-12-24

**Authors:** Vasvi Dhir, Isabel McDonald, Maude Gelinas Faucher, Ivan Sarmiento, Annick Gauthier, Neil Andersson, Mark J Yaffe, Maiya R. Geddes

**Affiliations:** ^1^ McGill University, Montreal, QC, Canada; ^2^ St Mary's Family Medicine Centre, Montreal, QC, Canada; ^3^ McGill University, Department of Family Medicine, Montreal, QC, Canada; ^4^ The Neuro, Faculty of Medicine, McGill University, Montreal, QC, Canada

## Abstract

**Background:**

Gender selection bias is a form of selection bias rooted in gender insensitivity or androcentrism, erroneously regarding research participants with different gender identities as similar/different. Neurocognitive aging research is largely subject to this bias, whereby study samples often overrepresent White, well‐educated, women living in less socioeconomically deprived areas. This biased representation raises cardinal concerns about health equity and discrimination and about generalizability and reproducibility of neuroscientific findings.

**Objectives:**

This study identified gender‐related barriers and facilitating factors older adults perceive when considering participation in observational neurocognitive aging research, as these factors may underly the bias.

**Method:**

We employed a participatory research methodology, called fuzzy cognitive mapping (FCM). The method is rooted in graph theory and social network analysis and portrays perspectives on what contributes to the occurrence of an outcome. Researchers discussed with participants from diverse groups and recorded their thoughts on what they perceived as factors encouraging or hindering their involvement in research. The factors were standardized across maps and organized in a causal network. We computed the fuzzy transitive closure (FTC) to quantify the maximum influence each factor has on other factors through direct and indirect links. The factors were aggregated into categories through inductive analysis. We computed measures of centrality, whereby categories with higher outdegree centrality scores were perceived as causes in the causal network and those with higher indegree centrality were perceived as outcomes. We operated analyses in a gender‐segregated manner.

**Results:**

We co‐created 24 maps with participants (12 women, 12 men). Our results show the shared and distinct factors older men and women perceive when considering participation in brain health research. Both men and women perceived individuals’ personal traits, quality of participant‐researcher communications, logistic considerations, and research‐specific practices as influential. Only women perceived their individual experiences related to the research as facilitating research engagement, whereas willingness to return benefits to the general population through research was exclusive to men.

**Conclusion:**

This study addressed the lack of diversity and poor representativeness, particularly of older men in neurocognitive aging research samples, to generate meaningful sampling strategies and ultimately knowledge suited to generalizable application, thus advancing toward unbiased science.